# Patterns of Sexual Practices, Sexually Transmitted Infections and Other Genital Infections in Women Who Have Sex with Women Only (WSWO), Women Who Have Sex with Men Only (WSMO) and Women Who Have Sex with Men and Women (WSMW): Findings from a Sexual Health Clinic in Melbourne, Australia, 2011–2019

**DOI:** 10.1007/s10508-022-02311-w

**Published:** 2022-07-01

**Authors:** Jaimie L. Engel, Christopher K. Fairley, Kate E. Greaves, Lenka A. Vodstrcil, Jason J. Ong, Catriona S. Bradshaw, Marcus Y. Chen, Tiffany R. Phillips, Eric P. F. Chow

**Affiliations:** 1 Melbourne Sexual Health Centre, Alfred Health, 580 Swanston Street, Carlton, VIC 3053 Australia; 2 Central Clinical School, Faculty of Medicine, Nursing and Health Sciences, Monash University, Melbourne, VIC Australia; 3 Centre for Epidemiology and Biostatistics, Melbourne School of Population and Global Health, The University of Melbourne, Melbourne, VIC Australia

**Keywords:** Heterosexual, Lesbian, Bisexual, Sexual health, Sexually transmitted diseases, Sexual orientation, Sexual activity, Sexual behavior

## Abstract

Despite rises in sexually transmitted infection (STI) notifications among Australian women in the last decade, limited STI surveillance data exist specifically for women who have sex with women. This study aimed to compare differences in sexual practices and positivity for STIs and other genital infections among women who have sex with men only (WSMO), women who have sex with women only (WSWO), and women who have sex with men and women (WSMW), and whether these changed over time. In this retrospective repeated cross-sectional study, women attending the Melbourne Sexual Health Centre for the first time between 2011 and 2019 were categorized as “WSMW,” “WSWO,” or “WSMO” according to self-reported sexual practices in the previous 12 months. Demographic information, sexual practices, and positivity for STIs and other genital infections were compared between the three groups and over time. A total of 36,147 women (2618 WSMW, 534 WSWO, and 32,995 WSMO) were included. WSMW reported more sexual partners (median = 6; IQR = 4–10) than WSMO (median = 3; IQR = 2–5) and WSWO (median = 2; IQR = 1–4) (*p* < .001). A higher proportion of WSMW always used condoms with casual male partners compared to WSMO (20.4% vs 15.9%; *p* < .001). The proportion of women who always used condoms with casual male partners decreased over time in WSMO, (19.9% in 2011 to 15.2% in 2019, *p*_trend_ < .001) but not in WSMW. Bacterial vaginosis was more common in WSWO (14.8%) than in WSMW (11.8%) and WSMO (7.7%) (*p* < .001). Chlamydia was more common in WSMO (9.3%) than in WSMW (6.6%) and WSWO (1.2%) (*p* < .001). Syphilis was more common in WSMO (1.0%) than in WSMW (0.3%) and WSWO (0.0%) (*p* = .004). Over time, chlamydia positivity in WSWO increased (from 0.0% to 2.7%, *p*_trend_ = .014), and syphilis positivity in WSMW increased (from 0.0% to 0.7%, *p*_trend_ = .028); however, positivity of these STIs did not change in other groups. Sexual practices and positivity for STIs and other genital infections differed according to the sex of women’s partners in the previous 12 months. Knowledge of these differences is important to account for future changes in STI trends that may occur in these subpopulations.

**Supplementary Information:**

The online version contains supplementary material available at 10.1007/s10508-022-02311-w.

## Introduction

Over the past decade in Australia, rates of sexually transmitted infections (STI) in women have been on the rise (Australian Institute of Health & Welfare, 2019). Between 2014 and 2019, the annual number of syphilis (defined in this context as being less than 2 years duration) notifications in women increased by almost 500% (from 164 to 962 cases) (Australian Government Department of Health, 2018). Over the same period, the annual number of gonorrhea notifications increased by over 120% (from 4200 to 9314 cases) (Australian Government Department of Health, 2021). Untreated STIs in women can have profound public health ramifications, leading to long-term reproductive sequelae such as chronic pelvic pain, pelvic inflammatory disease (PID), infertility and complications in pregnancy including spontaneous abortion, pre-term delivery, and neonatal infection (World Health Organization, 2019).

Currently in Australia, there are limited published STI data that examine the subpopulation of women who have sex with women (WSW). In contrast, extensive STI data exist for Australian men who have sex with men (MSM), and numerous epidemiological studies have established that in males, STI risk is higher among MSM compared with men who have sex with women only (Australian Government Department of Health, 2018; Chow et al., 2019a, 2019b; Jasek et al., 2017; Martin-Sanchez et al., 2020a, 2020b, 2020c). Given the lack of corresponding data in women, it is difficult to extrapolate whether similar patterns can be observed in Australian WSW, and if STI patterns or prevalence vary depending on whether women have sex with exclusively men, exclusively women, or both.

Numerous international studies have demonstrated appreciable risks of STIs among WSW, such as bacterial vaginosis, chlamydia, gonorrhea, herpes simplex virus, and human papillomavirus (Bailey et al., 2004; Gorgos & Marrazzo, 2011; Logie et al., 2015; Marrazzo et al., 2002; Molin et al., 2016; Xu et al., 2010). Several case studies and small reports have also described possible female–female transmission of syphilis (Campos-Outcalt & Hurwitz, 2002), trichomonas (Kellock & O'Mahony, 1996; Muzny et al., 2012), and HIV (Chan et al., 2014; Kwakwa & Ghobrial, 2003). These studies, however, are difficult to compare due to inconsistencies in the definition of WSW between studies, which may incorporate aspects of sexual identity, sexual practices, sexual orientation, or a combination thereof (Bauer & Brennan, 2013; Bauer & Jairam, 2008). Specifically, the tendency to group women who have sex with women only (WSWO) and women who have sex with men and women (WSMW) under the same umbrella of WSW raises difficulties in understanding the transmission dynamics of certain STIs among groups of women.

The aim of this study, therefore, was to analyze the sexual practices and positivity for STIs and other genital infections in WSW, and to determine whether differences exist between women who have sex with women only (WSWO), women who have sex with men and women (WSMW), and women who have sex with men only (WSMO) at a large, metropolitan sexual health center in Melbourne, Australia. These subgroups were specifically defined according to self-reported sexual practices within the previous 12 months. Additionally, we also aimed to examine whether temporal changes in sexual practices and positivity for STIs and other genital infections occurred between 2011 and 2019.

## Method

### Participants

We conducted a retrospective repeated cross-sectional study utilizing the electronic data of women who presented to the Melbourne Sexual Health Centre (MSHC) for the first time between 2011 and 2019. MSHC is a large, publicly funded sexual health clinic in metropolitan Melbourne that provided free STI testing, treatment, counseling, and other clinical services to clients on a walk-in basis during the study period.

Clients who identified as female, over 18 years of age, and who visited MSHC for the first time between 2011 and 2019 were eligible for inclusion in this analysis. We only included the client’s first visit to avoid any bias that may arise from frequently returning clients, as their sexual practices and STI risks may be different. Individuals who declined to report the number and gender of sexual partners in the previous 12 months were excluded from the analysis as they did not meet the criteria for classification into our three subgroups. Furthermore, individuals who reported current sex work on the day of visit were excluded from the analysis as their sexual practices and STI risks were different from women who were not sex workers (Chow et al., 2014, 2019b; Zappulla et al., 2020).

### Measures and Procedure

All clients were invited to complete a questionnaire using computer-assisted self-interview (CASI) on arrival to the clinic. CASI collects information regarding demographic characteristics (e.g., age, sex, country of birth, Aboriginal or Torres Strait Islander origin), sexual practices (e.g., gender, number, and type [regular or casual] of sexual partners, condom use with sexual partners) in the previous 12 months, and intravenous drug use in the previous 12 months.

As CASI does not ask clients to give their sexual identity or orientation, we categorized women into three groups according to self-reported sexual practices for this analysis. We defined “WSMO” as those who only had male partners in the previous 12 months, “WSWO” as those who had only female partners in the previous 12 months, and “WSMW” as those who had both male and female sexual partners in the previous 12 months.

Clients attending MSHC during the study period were offered testing for STIs and other genital infections depending on their sexual risk profile. Clients’ positivity for STIs and other genital infections were extracted from the clinic’s electronic database. We examined common laboratory-based STI diagnoses (chlamydia, gonorrhea, trichomonas, HIV, and syphilis), as well as clinically diagnosed symptomatic conditions in women (i.e., bacterial vaginosis [BV], candidiasis, herpes simplex virus [HSV], and pelvic inflammatory disease [PID]). For laboratory-based STI diagnoses, we defined positivity as the number of women who tested positive divided by the total number of women who had been tested for the infection. For clinically diagnosed symptomatic conditions, we defined positivity as the number of women who had a clinical diagnosis divided by the total number of women attending the clinic.

Both syphilis and HIV were diagnosed using serological testing. There was a change in the diagnostic method for chlamydia, gonorrhea, and trichomonas during the study period. Nucleic acid amplification tests (NAAT) were used for chlamydia diagnosis for the whole study period, however, we changed the diagnostic assay from the BD ProbeTec Strand Displacement Amplification Assay (Becton, Dickinson and Co, Sparks, Maryland, USA) to the Transcription-Mediated Amplification Aptima Combo 2 (AC2) Assay (Hologic Gen-Probe, San Diego, California, USA) in March 2015. We also changed the diagnostic method for gonorrhea and trichomonas from culture to NAAT using the AC2 assay in March 2015 and October 2018, respectively.

Before August 2017, asymptomatic screening for gonorrhea in women (except sex workers) was not recommended as per the Australian STI Management Guidelines (Australasian Sexual Health Alliance, 2019) and only women presenting with genital symptoms or who were self-reported contacts of a partner with gonorrhea were tested at MSHC. After August 2017, however, all women attending MSHC were offered screening for gonorrhea, regardless of the presence of symptoms (Martin-Sanchez et al., 2020a, 2020b, 2020c).

### Statistical Analysis

Demographic characteristics, sexual practices, and HIV/STI positivity were compared between the WSWO, WSMW, and WSMO groups using the chi-square test for categorical variables (e.g., condom use, STI positivity), or Kruskal–Wallis *H* test for continuous variables (e.g., age, number of sexual partners). If there were significant differences between the three groups (*p* < .05), sensitivity analyses using Mann–Whitney *U* test were performed to examine the differences between each pair of groups. For categorical variables, those who did not answer ‘yes’ or ‘no’ were excluded from the calculation of the *p* value in order to analyze only dichotomous answers, however, missing data were included in the tables to account for all women included in the study. Temporal analyses were conducted using the chi-square trend test for categorical variables (e.g., HIV/STI positivity) and the Jonckheere–Terpstra test for continuous variables (e.g., number of sexual partners). The 95% confidence intervals (CI) of the proportion were calculated using the binomial exact method.

Univariable and multivariable logistic regression analyses were performed to examine the association between positivity for STIs and other genital infections and sexual practices (i.e., WSMO, WSMW, and WSWO). Nine separate logistic regression models were run for each STI and genital infection (i.e., BV, candidiasis, HSV, PID, chlamydia, gonorrhea, trichomonas, HIV, and syphilis). The independent variables for each logistic regression remained constant and were chosen based on clinical knowledge and previous literature (Chowdhury & Turin, 2020), which included age, year of presentation, total number of sexual partners in the previous 12 months, country of birth (Rowley et al., 2019), and having sexual partners from overseas (outside of Australia) in the previous 12 months (Misson et al., 2018; Phillips et al., 2019). Crude and adjusted odds ratios (OR) and the corresponding 95% CI were reported.

Analyses were conducted using SPSS (version 26, Armonk, NY: IBM Corp) with the exception of the logistic regression models which were conducted using Stata (version 17, College Station, TX, USA).

## Results

**Fig. 1:**
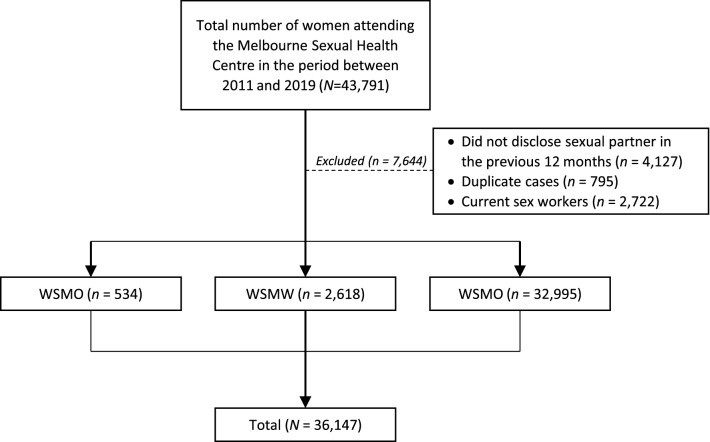
Flowchart outlining the selection and exclusion process for final analysis. N represents the total number of women and n represents the number of women in each subgroup

A total of 43,791 women attended the MSHC for the first time between 2011 and 2019 (Fig. 1). We excluded 7644 women, including 4127 women who did not disclose the number or sex of sexual partners in the previous 12 months, 2722 women who self-reported as current sex workers, and 795 women with duplicate electronic records from the same day. Hence, 36,147 women were included in the final analysis, consisting of 32,995 WSMO (91.3%), 2618 WSMW (7.2%), and 534 WSWO (1.5%) women.

### Demographic Characteristics

During the study period, the overall number of women presenting to MSHC increased across all three groups (Table 1), however, the proportions of the groups changed considerably. The proportion of WSWO and WSMO presenting to MSHC decreased over time (from 2.1% in 2011 to 1.4% in 2019, *p*_trend_ = .008 for WSWO, and from 93.0% in 2011 to 89.2% in 2019, *p*_trend_ < 001 for WSMO), while the proportion of WSMW increased significantly from 4.9% in 2011 to 9.4% in 2019 (*p*_trend_ < .001).

**Table 1:** Temporal analysis of the number and proportion of WSWO, WSMW, and WSMO attending the Melbourne Sexual Health Clinic between 2011 and 2019

	2011	2012	2013	2014	2015	2016	2017	2018	2019	Total	*p*_trend_
WSWO	55 (2.1%)	49 (1.6%)	63 (1.7%)	64 (1.7%)	52 (1.3%)	50 (1.2%)	50 (1.2%)	74 (1.5%)	77 (1.4%)	534 (1.5%)	.008
WSMW	128 (4.9%)	191 (6.1%)	200 (5.5%)	239 (6.3%)	261 (6.6%)	344 (7.9%)	362 (8.7%)	369 (7.6%)	524 (9.4%)	2,618 (7.2%)	< .001
WSMO	2416 (93.0%)	2892 (92.3%)	3398 (92.8%)	3501 (92.0%)	3649 (92.1%)	3949 (90.9%)	3769 (90.1%)	4443 (90.9%)	4978 (89.2)	32,995 (91.3%)	< .001
Total	2599	3132	3661	3804	3962	4343	4181	4886	5579	36,147	

There was no significant difference in median age between WSMO (25 years, IQR: 23–29) and WSMW (25 years, IQR: 22–29) (*p* = .200), however, WSWO were older (27 years, IQR: 23–31) than both WSMO (*p* < .001) and WSMW (*p* < .001) (Table 2). Overall, 23,552 women (65.2%) were born overseas; more WSMO were born overseas (66.6%), followed by WSMW (53.1%) and then WSWO (37.3%) (*p* < .001). For all women who were born overseas, the top three regions of birth were the UK and Ireland (20.3%, *n* = 7326), the USA (3.9%, *n* = 1410), and France (3.7%, *n* = 1332). A total of 318 women (0.9%) self-identified as being of Aboriginal or Torres Strait Islander origin, and this proportion did not differ between groups (*p* = .480).

**Table 2:** Comparison of demographic characteristics and sexual practices of WSWO, WSMW, and WSMO attending the Melbourne Sexual Health Clinic between 2011 and 2019

	WSWO (N = 534)	WSMW (N = 2,618)	WSMO (N = 32,995)	*p* value (WSWO, WSMW, and WSMO)	*p* value (WSWO with WSMW)	*p* value (WSWO with WSMO)	*p* value (WSMW with WSMO)
Median age (years), (IQR)	27, (23–31)	25, (22–29)	25, (23–29)	< .001	< .001	< .001	.200
Country of birth				< .001	< .001	< .001	< .001
Australia	312 (58.4%)	1,127 (43.0%)	9,634 (29.2%)				
Overseas	199 (37.3%)	1,391 (53.1%)	21,963 (66.6%)				
No information given*	23 (4.3%)	100 (3.8%)	1,398 (4.2%)				
Aboriginal or Torres Strait Islander Origin				.480			
Yes	6 (1.1%)	29 (1.1%)	283 (0.9%)				
No	487 (91.2%)	2,390 (91.3%)	28,927 (87.7%)				
No information given*	41 (7.7%)	199 (7.6%)	3,785 (11.5%)				
Intravenous drug use in the previous 12 months				< .001	.305	< .001	< .001
Yes	11 (2.1%)	38 (1.5%)	205 (0.6%)				
No	519 (97.2%)	2,552 (97.5%)	32,537 (98.6%)				
No information given*	4 (0.7%)	28 (1.1%)	253 (0.8%)				
Number of total sexual partners in the previous 12 months, median (IQR)	2 (1–4)	6 (4–10)	3 (2–5)	< .001	< .001	< .001	< .001
Number of female sexual partners in the previous 12 months, median (IQR)	2 (1–4)	1 (1–2)	N/A	N/A	< .001	N/A	N/A
Number of male sexual partners in the previous 12 months, median (IQR)	N/A	5 (2–8)	3 (2–5)	N/A	N/A	N/A	< .001
Current regular sexual partner *(n,* [%])				< .001	< .001	< .001	.220
Yes	311 (58.2%)	1,165 (44.5%)	14,598 (44.2%)				
No	185 (34.6%)	1,394 (53.2%)	16,605 (50.3%)				
No information given	38 (7.1%)	59 (2.3%)	1,792 (5.4%)				
Current regular sexual partner, stratified by the gender of the partner (*n,* [%])*				N/A			
No regular partner	185 (34.6%)	1,394 (53.2%)	16,605 (50.3%)				
Regular female partner	311 (58.2%)	233 (8.9%)	N/A				
Regular male partner	N/A	854 (32.6%)	14,598 (44.3%)				
Regular male and female partner	N/A	78 (3.0%)	N/A				
No information given	38 (7.1%)	59 (2.3%)	1,792 (5.4%)				
Condom use with current regular male sexual partner in the previous 12 months (*n,* [%])	–	–	–	.452			
Always	N/A	159 (17.1%)	2,633 (18.0%)				
Not always	N/A	773 (82.9%)	11,965 (82.0%)				
Casual sexual partner(s) in the previous 12 months*				< .001	< .001	< .001	< .001
Yes	426 (79.8%)	2,496 (95.3%)	27,618 (83.7%)				
No	88 (16.5%)	61 (2.3%)	3,292 (10.0%)				
No information given	20 (3.7%)	61 (2.3%)	2,085 (6.3%)				
Casual sexual partner(s) in the previous 12 months, stratified by the gender of the partner (*n,* [%])*				N/A			
No casual partner(s)	88 (16.5%)	61 (2.3%)	3,292 (10.0%)				
Casual female partner(s)	426 (79.8%)	190 (7.3%)	N/A				
Casual male partner(s)	N/A	66 (2.5%)	27,618 (83.7%)				
Casual male and female partner(s)	N/A	2,240 (85.6%)	N/A				
No information given	20 (3.7%)	61 (2.3%)	2,085 (6.3)				
Condom use with casual male sexual partner(s) in the previous 12 months (*n,* [%])	–	–	-	< .001			
Always	N/A	470 (20.4%)	4,399 (15.9%)				
Not always	N/A	1,836 (79.6%)	23,219 (84.1%)				

*Women with missing data excluded from calculation of *p* value in order to analyze only dichotomous answers, however still included in tables to account for all women included in the study

### Sexual and Drug Use Practices

WSMW had the highest median number of sexual partners in the previous 12 months (6, IQR: 4–10), followed by WSMO (3, IQR: 2–5) and WSWO (2, IQR: 1–4) (Table 2) (*p* < .001), and the median number of sexual partners increased over time for all groups (WSMW: median 6 to 7, *p*_trend_ < .001; WSMO: median 2 to 3, *p*_trend_ < .001; WSWO: median 2 to 3, *p*_trend_ = .062) (Table S1). WSMW had more male sexual partners than WSMO (median 5 vs 3, *p* < .001), however, WSMW had fewer female sexual partners than WSWO (median 1 vs 2, *p* < .001). Of the 2618 WSMW, 2039 (77.9%) had more male than female partners, 315 (12.0%) had more female than male partners, and 264 (10.1%) had equal numbers of male and female partners.

**Fig. 2:**
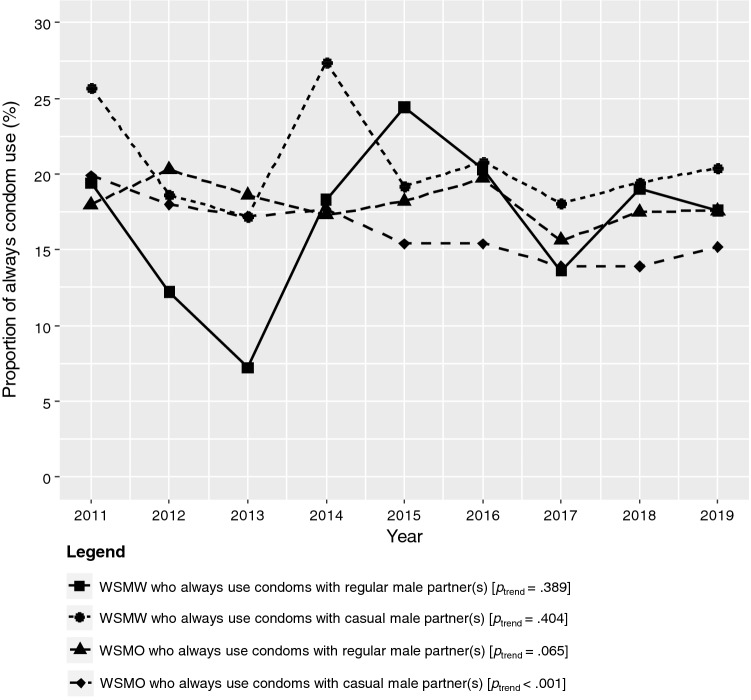
The proportion of WSMW and WSMO who always use condoms for vaginal or anal sex with their regular and casual male partners in the previous 12 months between 2011 and 2019

Of all three groups, more WSWO reported having a current regular sexual partner (58.2%), compared to WSMW (44.5%) and WSMO (44.2%). There was no significant difference in the proportion of women who always used condoms with current regular male partners for vaginal or anal sex between WSMW (17.1%) and WSMO (18.0%) (*p* = .452), and these proportions did not change significantly over time (Fig. 2).

More WSMW reported having a casual sexual partner in the previous 12 months (95.3%), followed by WSMO (83.7%) and then WSWO (79.8%) (*p* < .001). The proportion of women who always used condoms with casual male partners for vaginal or anal sex in the previous 12 months was higher in WSMW (20.4%) compared with WSMO (15.9%) (*p* < .001). The proportion of women who always used condoms with casual male partners in the previous 12 months decreased significantly in WSMO, from 19.9% in 2011 to 15.2% in 2019 (*p*_trend_ < .001), but it did not change significantly in WSMW (*p*_trend_ = .404) (Fig. 2).

The proportion of women who engaged in intravenous drug use in the previous 12 months was highest in WSWO (2.1%), followed by WSMW (1.5%) and then WSMO (0.6%) (*p* < .001). The proportion of women who engaged in intravenous drug use did not change significantly over time in WSMW (*p*_trend_ = .520), WSWO (*p*_trend_ = .058), and WSMO (*p*_trend_ = .826) (Table S2).

### Positivity for STIs and Other Genital Infections

BV was the most common infection among all three groups of women (Table 3): Diagnosis of BV was highest among WSWO (14.8%), followed by WSMW (11.8%) and then WSMO (7.7%) (*p* < .001). The reverse trend was seen for chlamydia, with WSMO having the highest positivity (9.3%), followed by WSMW (6.6%) and then WSWO (1.2%) (*p* < .001). Additionally, there was a significant increase in chlamydia positivity in WSWO from 0.0% in 2011 to 2.7% in 2019 (*p*_trend_ = .014) (Table S2), however, chlamydia positivity did not change significantly in WSMW and WSMO. Similarly, WSMO had the highest syphilis positivity (1.0%) followed by WSMW (0.3%) and then WSWO (0.0%) (*p* = .004). There was a significant increase in syphilis positivity among WSMW (from 0.0% in 2011 to 0.7% in 2019, *p*_trend_ = .028) but syphilis remained stable over time in WSMO and non-existent in WSWO. PID diagnosis was lowest in WSWO (0.4%) compared to WSMW (2.5%) and WSMO (2.5%) (*p* = .007). Among all women, 8.7% were diagnosed with candidiasis, 3.2% were diagnosed with HSV, 1.1% were diagnosed with gonorrhea, 0.6% were diagnosed with trichomonas, and 0.3% were diagnosed with HIV. These proportions did not differ significantly across all three groups. After adjustment for confounding factors, WSWO and WSMW had a higher odds of BV positivity in comparison to WSMO (WSWO: aOR 2.4, 95% CI 1.8–3.0 [*p* < .001]; WSMW: aOR 1.5, 95% CI 1.3–1.7 [*p* < .001]). In contrast, WSWO and WSMW had a lower odds of chlamydia positivity compared to WSMO (WSWO: aOR 0.2, 95% CI 0.1–0.3 [*p* < .001]; WSMW: aOR 0.6, 95% CI 0.5–0.7 [*p* < .001]). In comparison to WSMO, WSWO had lower odds of PID positivity (aOR 0.1, 95% CI 0.0–0.6 [*p* = .007]) and WSMW had lower odds of syphilis (aOR 0.4, 95% CI 0.2–0.9 [*p* = .027]) in the adjusted analysis. There were no other significant differences in the odds of positivity for other STIs/genital infections between the three groups in the adjusted analysis (Table 4).

**Table 3:** Comparison of STIs and other genital infections among WSMW, WSWO, and WSMO attending the Melbourne Sexual Health Clinic between 2011 and 2019

	WSWO (N = 534)			WSMW (N = 2618)			WSMO (N = 32,995)			*p* value (WSWO, WSMW and WSMO)	*p* value (WSMW and WSWO)	*p* value (WSWO and WSMO)	*p* value (WSMW and WSMO)
	Number of women tested (N)	Number of positive cases (n)	% positive cases (95% CI)	Number of women tested (N)	Number of positive cases (n)	% positive cases (95% CI)	Number of women tested (N)	Number of positive cases (n)	% positive cases (95% CI)				
Bacterial Vaginosis	534	79	14.8 (11.9–18.1)	2,618	309	11.8 (10.6–13.1)	32,995	2,540	7.7 (7.4–8.0)	< .001	.055	< .001	< .001
Candidiasis	534	41	7.7 (5.6–10.3)	2,618	210	8.0 (7.0–9.1)	32,995	2,886	8.7 (8.4–9.1)	.317	.789	.205	.385
Herpes simplex virus	534	19	3.6 (2.2–5.5)	2,618	84	3.2 (2.6–4.0)	32,995	1,071	3.2 (3.1–3.4)	0.915	.679	.917	.687
Pelvic inflammatory disease	534	2	0.4 (0.0–1.4)	2,618	65	2.5 (1.9–3.2)	32,995	832	2.5 (2.4–2.7)	.007	.002	.002	.903
Chlamydia	488	6	1.2 (0.5–2.7)	2,534	167	6.6 (5.7–7.6)	30,811	2,851	9.3 (8.9–9.6)	< .001	< .001	< .001	< .001
Gonorrhea^*^	333	1	0.3 (0.0–1.7)	1,878	20	1.1 (0.7–1.6)	20,187	238	1.2 (1.0–1.3)	.307	.185	.660	.138
Culture	103	0	0 (0.0–3.5)	400	4	1.0 (0.3–2.5)	5,379	41	0.8 (0.6–1.0)	.581	.308	.602	.374
NAAT ± culture	230	1	0.4 (0.0–2.4)	1,478	16	1.1 (0.6–1.8)	14,808	197	1.3 (1.2–1.5)	.368	.357	.424	.237
Trichomonas ^†^	150	2	1.3 (0.2–4.7)	730	7	1.0 (0.4–2.0)	7,709	41	0.5 (0.4–0.7)	.166	.678	.143	.188
HIV	264	0	0 (0–1.4)	1,809	1	0.1 (0.0–0.3)	18,704	67	0.4 (0.3–0.5)	.063	.702	.032	.330
Syphilis	268	0	0 (0–1.4)	1,811	6	0.3 (0.1–0.7)	18,815	195	1.0 (0.9–1.2)	.004	.345	.004	.094

*NAAT* nucleic acid amplification tests, *HIV* human immunodeficiency virus

* During the period between March 2015 and 2019, 12 women in total were tested for gonorrhea by culture only instead of NAAT. This was because these women had NAAT performed outside MSHC and were then referred to MSHC for a confirmatory culture and treatment. As such, these women were included under the “NAAT ± culture” category. Additionally, between March 2015 and 2019, 6 women in total were tested for gonorrhea by NAAT and culture with an invalid NAAT result but a positive culture. We considered these 6 women as positive cases based on their positive culture results and were included under the “NAAT ± culture” category

^†^ The diagnostic method for trichomonas was changed from culture to NAAT using transcription-mediated amplification Aptima Combo 2 (AC2) Assay (Hologic Gen-Probe, San Diego, California, USA) in October 2018

**Table 4:** Association between sexual practices among women attending the Melbourne Sexual Health Clinic between 2011 and 2019 and positivity for BV, candidiasis, HSV, PID, chlamydia, gonorrhea, trichomonas, HIV, and syphilis

HIV/STI	OR (95% CI)	*p* value	aOR (95% CI)*	*p* value
Bacterial vaginosis				
WSMO^‡^	1	ref	1	ref
WSMW^§^	1.6 (1.4 – 1.8)	< .001	1.5 (1.3 – 1.7)	< .001
WSWO^¶^	2.1 (1.6 – 2.7)	< ..001	2.4 (1.8 – 3.0)	< .001
Candidiasis				
WSMO	1	ref	1	ref
WSMW	0.9 (0.8 – 1.1)	.205	1.0 (0.8 – 1.1)	.629
WSWO	0.9 (0.6 – 1.2)	.386	0.9 (0.6 – 1.2)	.359
Herpes Simplex Virus				
WSMO	1	ref	1	ref
WSMW	1.0 (0.8 – 1.2)	.917	1.0 (0.8 – 1.2)	.862
WSWO	1.1 (0.7 – 1.7)	.687	1.0 (0.6 – 1.5)	.833
Pelvic Inflammatory Disease				
WSMO	1	ref	1	ref
WSMW	1.0 (0.8 – 1.3)	.903	0.9 (0.7 – 1.2)	.643
WSWO	0.1 (0.0 – 0.6)	.007	0.1 (0.0 – 0.6)	.007
Chlamydia				
WSMO	1	ref	1	ref
WSMW	0.7 (0.6 – 0.8)	< .001	0.6 (0.5 – 0.7)	< .001
WSWO	0.1 (0.1 – 0.3)	< .001	0.2 (0.1 – 0.3)	< .001
Gonorrhea				
WSMO	1	ref	1	ref
WSMW	0.9 (0.6 – 1.4)	.660	0.8 (0.5 – 1.3)	.305
WSWO	0.3 (0.0 – 1.8)	.170	0.2 (0.0 – 1.5)	.117
Trichomonas				
WSMO	1	ref	1	ref
WSMW	1.8 (0.8 – 4.1)	.148	1.7 (0.7 – 4.1)	.232
WSWO	2.5 (0.6 – 10.5)	.203	2.1 (0.5 – 9.1)	.303
HIV				
WSMO	1	ref	1	ref
WSMW	0.2 (0.0 – 1.1)	.063	0.2 (0.0 – 1.5)	.111
WSWO	–†	–†	–†	–†
Syphilis				
WSMO	1	ref	1	ref
WSMW	0.3 (0.1 – 0.7)	.006	0.4 (0.2 – 0.9)	.027
WSWO	–^†^	–^†^	–^†^	–^†^

*Adjusted for age, year, total number of sexual partners, country of birth, and having sexual partners from overseas (outside of Australia)

^†^There were no WSWO who were diagnosed with HIV or syphilis in our study

^‡^WSMO refers to ‘women who have sex with men only’

^§^WSMW refers to ‘women who have sex with men and women’

^¶^WSWO refers to ‘women who have sex with women only’

## Discussion

In this study of 36,147 women attending the MSHC between 2011 and 2019, we found significant differences in sexual practices and diagnoses of STIs and other genital infections among WSMW, WSWO, and WSMO, as well as significant changes over time. Our major findings included a substantial increase in the proportion of WSMW attending MSHC, as well as an increase in median partner numbers over time for all three groups. Additionally, WSMW were found to have more sexual partners, but also more frequent condom use compared to WSMO. In terms of STIs and other genital infections, BV was most common in WSWO and least common in WSMO, whereas the inverse was true for infections such as chlamydia, PID, HIV, and syphilis, which were least common in WSWO and most common in WSMO. Interestingly, for most STIs and other genital infections, the proportions of WSMW diagnosed appeared to be in the middle of the proportions observed in WSWO and WSMO, suggesting that WSMW could be at risk of acquiring and transmitting STIs classically seen in either WSWO or WSMO. Ultimately, this study is one of the largest Australian studies to date to directly compare WSMO, WSMW, and WSWO, and highlights that these groups of women are unique in their sexual health needs and risk factors.

BV diagnoses were found to be highest in WSWO, followed by WSMW and then WSMO. Similar results were found in a recent American study examining BV among African-American women according to the sex of sexual partners in the previous 12 months, (Olson et al., 2018), with a higher likelihood of acquiring BV in WSWO (aOR 2.63, 95% CI 1.55–4.48) and WSMW (aOR: 3.67, 95% CI 2.17–6.21) in comparison to WSMO. This study also classified WSW according to the same criteria used in the present study. Numerous previous studies have also demonstrated a high prevalence of BV among WSW in general, however, definitions of WSW have varied between studies (Evans et al., 2007; Fethers et al., 2008; Forcey et al., 2015). The high prevalence of BV among WSW may support the notion of the exchange of vaginal bacterial species during female–female sexual interaction, and the concordance of vaginal bacterial flora that is often found among female–female partnerships (Bradshaw et al., 2014; Marrazzo et al., 2002). Our data additionally demonstrated an increase in BV diagnoses among WSMO over time. Past studies have provided evidence supporting the carriage of BV-associated bacteria in men (Schwebke et al., 2014; Zozaya et al., 2016), and our data additionally demonstrated reductions in condom use among WSMO with their male partners; a known risk factor for BV according to a 2008 meta-analysis (Fethers et al., 2008). These factors in combination may be driving the increasing positivity of BV seen in WSMO. A randomized clinical trial is currently underway to examine the role of concurrent male partner treatment of BV (Plummer et al., 2021; Vodstrcil et al., 2020).

Since the primary means of transmission of genital infections such as chlamydia, gonorrhea, syphilis, and HIV in women is via penile–vaginal sex (Australian Government Department of Health, 2012), we would expect positivity to be higher in those who have had male sexual partners (i.e., WSMO and WSMW rather than WSWO). This pattern was demonstrated in our study, and has also been observed in numerous other studies (Bailey et al., 2004; Marrazzo et al., 2001; Molin et al., 2016), although it is important to note that the definitions of WSW may have differed between studies. While recent sex with men is strongly associated with STI positivity among WSW (Bailey et al., 2004; Logie et al., 2015; Marrazzo et al., 2001; Muzny et al., 2011), this does not mean that WSWO are at a negligible risk of STIs. A study by Bauer et al. (Bauer & Welles, 2001) found that 13% of WSWO had a history of STIs, and an adjusted analysis highlighted that when controlled for female–male sexual activity, the frequency of female–female sexual interactions was independently associated with an increased odds of STI. A 2019 systematic review examining STI prevalence among self-identified lesbian women and/or women who have sex exclusively with women (Takemoto et al., 2019) estimated a 4.9 – 37.8% lifetime prevalence of any STI. That being said, there are a number of key concerns preventing accurate estimation of STI risk among WSWO—both in the present study and in wider literature. The first surrounds the definition of WSWO according to partners within the previous 12 months, which may inadvertently capture a cohort of women who, although had only reported female partners in the previous 12 months, may have had sex with a man more than 12 months prior. Since it is known that some STIs, such as chlamydia, (Molin et al., 2016; Price et al., 2016), can remain asymptomatic for more than 12 months in women, it is plausible that WSWO could have acquired these STIs from previous male partners. Second, the classification of WSW in general within the wider literature is markedly varied (Bauer & Brennan, 2013; Bauer & Jairam, 2008; Young & Meyer, 2005), encompassing aspects of sexual practices, sexual orientation, or both. This makes comparisons between studies and analyses of trends very difficult. Finally, there are little data that quantify the risk of female–female STI transmission or delineates risk according to specific sexual practices (i.e., sex toy use, digital–vaginal sex, oral–vaginal sex, oral–anal sex, direct vulval contact). The CASI questionnaire in our study only collected data on penile–vaginal sex but not these specific sexual practices, hence, we could not determine if associations existed between patterns of sexual practices and STI positivity or if changes in sexual practices occurred over time that could explain temporal changes in STI positivity. Both of these issues represent key deficits in current literature and potential opportunities for future research.

In our study, WSMW had a higher median number of male sexual partners in comparison to WSMO (median = 5 in WSMW vs median = 3 in WSMO), which is a known risk factor for STI positivity among WSW and is consistent with the results of previous studies (Bauer & Welles, 2001; Eisenberg, 2001; Gonzalez et al., 1999; Logie et al., 2015; Muzny et al., 2014). However, despite having more male partners, WSMW still had a lower positivity of genital infections classically transmitted via penile–vaginal sex, such as chlamydia, syphilis, gonorrhea, and HIV, when compared to WSMO. This could relate to our finding of higher proportions of condom use in WSMW than in WSMO, which could play a protective role against the acquisition of these infections. Despite these findings, we still observed that the overall proportions of consistent condom use in both groups was low when compared to national data, especially in regard to casual male partners. The Debrief Survey (Adam et al., 2019), a national survey conducted among young Australians aged 15–29 years old in 2019, reported that 48.0% of female respondents always used condoms with their casual male partners in the previous 12 months. Similarly, the Second Australian Study of Health and Relationships (ASHR2), a national population-based study conducted in 2012–2013 (de Visser et al., 2014), found that 49.4% of all women always used condoms with casual male partners in the previous 6 months, whereas our study demonstrated that only 20.4% of WSMW and 15.9% of WSMO (which equates to 16.3% of women with casual male partners overall) always used condoms with casual male partners in the previous 12 months. Similarly, these discrepancies may be explained by the fact that our study was conducted at a sexual health clinic, whereas the ASHR2 and the Debrief Survey were a population-based surveys; since the reasons for attending a sexual health clinic are likely related to the presence of symptoms or potential infection exposures, condom use is likely to be less frequent among those who attend a sexual health clinic than the general population.

It is important to note that we only collected data on condom use with male partners, and therefore condom use data are only applicable to WSMW and WSMO, but not WSWO. Since we did not collect any data on alternate barriers for STI prevention (i.e., dental dams), it was not possible to ascertain whether they were used during female-to-female sexual activities. Past studies, however, have found that the use of dental dams is uncommon among women (Bailey et al., 2003; Grulich et al., 2014; Richters & Clayton, 2010). A Sydney-based survey conducted 2004 (Richters et al., 2010) found that only 9.7% of WSW had used a dental dam during same-sex sexual activities in the previous 6 months.

Our findings pertaining to intravenous drug use reflected the results of numerous Australian and international studies (Fethers et al., 2000; Gonzalez et al., 1999; Mercer et al., 2007; Scheer et al., 2002), namely that WSW in general were more likely to use intravenous drugs compared to WSMO. However, these data must be interpreted with caution as the number of women who reported intravenous drug use was small in our study (*n* = 254). Similar patterns can also be observed in studies defining WSW according to sexual orientation (i.e., lesbian, bisexual, or heterosexual); a 2013 Australian population-based study (Roxburgh et al., 2016) demonstrated more than a fourfold higher odds of intravenous drug use among lesbian and bisexual women in comparison to heterosexual women, and similar patterns can be observed when comparing gay and bisexual men to heterosexual men (Martin-Sanchez et al., 2020a, 2020b, 2020c; Phillips et al., 2019). These findings may be due to stigmatization associated with sexual minority status and social norms in the LGBTQIA + community (Corliss et al., 2006). A higher proportion of intravenous drug use among WSW (defined according to sexual practices and/or sexual orientation) prompts consideration of whether these populations are at an increased risk of contracting blood-borne viruses, such as HIV and hepatitis C.

This study has several limitations. First, we defined WSMW, WSWO, and WSMO groups according to self-reported sexual practices in the previous 12 months, and it is important to note that sexual practices may not necessarily correlate with sexual orientation (Bauer & Jairam, 2008; Everett, 2013; Young & Meyer, 2005). Therefore, the findings from this study may not be generalizable to self-identified lesbian, bisexual, or heterosexual women, however, may provide insight for women who identify with these sexual orientations and also engage in the sexual practices outlined in this study. Second, our definition of WSMW by virtue relied on the individual having at least two sexual partners in the previous 12 months (one male and one female), whereas WSMO and WSWO only required one male and one female partner, respectively, to be defined as such. This could bias the population of WSMW toward those with more sexual partners, which is associated with a greater risk of STI acquisition and transmission. Interestingly, however, our study found no STIs or other genital infections in which the positivity was highest among WSMW. Third, this study was conducted at a single sexual health clinic in metropolitan Melbourne, and therefore may be subject to selection bias toward women who are more likely to have STIs since the primary reason for presentation to a sexual health center is usually due to the presence of STI symptoms. As such, our results may not be generalizable to the population of women Australia-wide, although changes we observed over time and between groups of women are valid. Fourth, limitations may have arisen due to the changes in gonorrhea screening policies introduced in August 2017 to include asymptomatic women. Prior to this time, only women who were symptomatic or who were contacts of a known gonorrhea case were offered gonorrhea screening at MSHC, meaning that a large number of asymptomatic women may have been missed. This is supported by a 2020 studying highlighting that up to half of cases of gonorrhea in women are asymptomatic (Martin-Sanchez et al., 2020a, 2020b, 2020c). Finally, limitations may have arisen due to the low numbers of WSWO in comparison to WSMO. Although there were only 534 (1.5%) WSWO in our study, this proportion is considerably higher than national estimates (0.1% in 2002, 0.3% in 2012, and 0.6% in 2017) (Australian Longitudinal Study on Women's Health, 2019; Richters et al., 2014). The small sample size and the infrequent cases of STIs may have limited the statistical power to detect differences between groups, and hence caution should be taken when interpreting STI trends in this group over time.

### Conclusion

This study highlighted that the sexual practices and positivity for STIs and other genital infections differ according to the sex of women’s sexual partners. We found that certain genital infections were more common in WSWO (e.g., BV) and WSMO (e.g., chlamydia), and that the STI positivity in WSMW appeared to occupy a midpoint between WSWO and WSMO. Sexual practices were heterogeneous between groups, however, overall proportions of condom use were low and the median number of sexual partners increased for all groups.

Currently, few STI prevention campaigns target specific subgroups of women according to the sex of their sexual partners. Future STI prevention campaigns and HIV/STI screening recommendations should therefore take this into consideration and be tailored according to the specific sexual practices of women. Scaling up of safe-sex and STI education for women who have sex with women, as well as further research into specific female–female sexual practices could also prove useful in the prevention and control of the rising rates of STIs among women in Australia.

## Supplementary Information

Below is the link to the electronic supplementary material.

Supplementary file1 (DOCX 42 kb)

## Data Availability

All relevant data are included in this manuscript.
