# Supplementary material for: Patterns of Sexual Practices, Sexually Transmitted Infections and Other Genital Infections in Women Who Have Sex with Women Only (WSWO), Women Who Have Sex with Men Only (WSMO) and Women Who Have Sex with Men and Women (WSMW): Findings from a Sexual Health Clinic in Melbourne, Australia, 2011–2019
Source: Arch Sex Behav. 2022 Jul 1;51(5):2651–65. doi: 10.1007/s10508-022-02311-w (PMC9293838; doi:10.1007/s10508-022-02311-w)
Supplement: Supplementary file 1 — Supplementary file1 (DOCX 42 kb) [file 10508_2022_2311_MOESM1_ESM.docx]

# **Supplementary Tables**

***Supplementary Table 1: Temporal analysis of the sexual and drug use practices of WSMW, WSWO and WSMO attending the Melbourne Sexual Health Clinic from 2011-2019***

| **Populations** |  | **2011** | **2012** | **2013** | **2014** | **2015** | **2016** | **2017** | **2018** | **2019** | ***p*_trend_** |
| --- | --- | --- | --- | --- | --- | --- | --- | --- | --- | --- | --- |
| **WSMW** | **Intravenous drug use in the previous 12 months (*n* [IQR])** | | | | | | | | | | 0.520 |
|  | **Yes** | 1 (0.8) | 7 (3.7) | 2 (1.0) | 1 (0.4) | 6 (2.3) | 4 (1.2) | 5 (1.4) | 5 (1.4) | 7 (1.4) |  |
|  | **No** | 127 (99.2) | 183 (96.3) | 196 (99.0) | 234 (99.6) | 254 (97.7) | 337 (98.8) | 351 (98.6) | 362 (98.6) | 508 (98.6) |  |
|  | **Median number of sexual partners in the previous 12 months (*n* [IQR])** | | | | | | | | | | <0.001 |
|  |  | 6 (4-9.75) | 6 (4-9) | 6 (4-9) | 6 (4-9) | 6 (4-10) | 7 (4-10) | 7 (4-10) | 7 (4-10) | 7 (4-11) |  |
|  | **Condom use with current regular male sexual partner(s) in the previous 12 months (*n* [%])** | | | | | | | | | | 0.389 |
|  | **Always** | 13 (19.4) | 9 (12.2) | 6 (7.2) | 17 (18.3) | 21 (24.4) | 25 (20.3) | 17 (13.6) | 20 (19.0) | 31 (17.6) |  |
|  | **Not always** | 54 (80.6) | 65 (87.8) | 77 (92.8) | 76 (81.7) | 65 (75.6) | 98 (79.7) | 108 (86.4) | 85 (81.0) | 145 (82.4) |  |
|  | **Condom use with casual male sexual partner(s) in the previous last 12 months (*n* [%])** | | | | | | | | | | 0.404 |
|  | **Always** | 27 (25.7) | 31 (18.6) | 30 (17.2) | 57 (27.4) | 44 (19.2) | 64 (20.8) | 59 (18.1) | 63 (19.4) | 95 (20.4) |  |
|  | **Not always** | 78 (74.3) | 136 (81.4) | 144 (82.8) | 151 (72.6) | 185 (80.8) | 243 (79.2) | 267 (81.9) | 261 (80.6) | 371 (79.6) |  |
| **WSWO** | **Intravenous drug use in the previous 12 months (*n* [IQR])** | | | | | | | | | | 0.058 |
|  | **Yes** | 4 (7.4) | 0 (0.0) | 3 (4.8) | 0 (0.0) | 0 (0.0) | 1 (2.0) | 2 (4.1) | 1 (1.4) | 0 (0.0) |  |
|  | **No** | 50 (92.6) | 49 (100.0) | 60 (95.2) | 64 (100.0) | 52 (100.0) | 49 (98.0) | 47 (95.9) | 73 (98.6) | 75 (100.0) |  |
|  | **Median number of sexual partners in the previous 12 months (*n* [IQR])** | | | | | | | | | | 0.062 |
|  |  | 2 (1-4) | 2 (1-4) | 3 (1-4) | 2 (1-3.75) | 2 (1-3) | 3 (1-4.25) | 3 (2-5) | 2.5 (2-4) | 3 (1-5) |  |
| **WSMO** | **Intravenous drug use in the previous 12 months (*n* [IQR])** | | | | | | | | | | 0.826 |
|  | **Yes** | 20 (0.8) | 18 (0.6) | 19 (0.6) | 29 (0.8) | 21 (0.6) | 11 (0.3) | 13 (0.3) | 41 (0.9) | 33 (0.7) |  |
|  | **No** | 2,380 (99.2) | 2,860 (99.4) | 3,367 (99.4) | 3,446 (99.2) | 3,601 (99.4) | 3,915 (99.7) | 3,718 (99.7) | 4,355 (99.1) | 4,895 (99.3) |  |
|  | **Median number of sexual partners in the previous 12 months (*n* [IQR])** | | | | | | | | | | <0.001 |
|  |  | 2 (1-4) | 3 (2-4) | 3 (2-5) | 3 (2-5) | 3 (2-5) | 3 (2-5) | 3 (2-5) | 3 (2-5) | 3 (2-5) |  |
|  | **Condom use with current regular male sexual partner(s) in the previous 12 months (*n* [%])** | | | | | | | | | | 0.065 |
|  | **Always** | 212 (18.0) | 285 (20.3) | 304 (18.6) | 285 (17.3) | 289 (18.2) | 331 (19.7) | 244 (15.6) | 329 (17.5) | 354 (17.6) |  |
|  | **Not always** | 965 (82.0) | 1,119 (79.7) | 1,331 (81.4) | 1,361 (82.7) | 1,303 (81.8) | 1,352 (80.3) | 1,318 (84.4) | 1,553 (82.5) | 1,663 (82.4) |  |
|  | **Condom use with casual male sexual partner(s) in the previous 12 months (*n* [%])** | | | | | | | | | | <0.001 |
|  | **Always** | 369 (19.9) | 426 (18.0) | 482 (17.2) | 513 (17.7) | 476 (15.4) | 522 (15.4) | 444 (13.9) | 525 (13.9) | 642 (15.2) |  |
|  | **Not always** | 1,488 (80.1) | 1,936 (82.0) | 2,317 (82.8) | 2,392 (82.3) | 2,610 (84.6) | 2,877 (84.6) | 2,747 (86.1) | 3,264 (86.1) | 3,588 (84.8) |  |

***Supplementary Table 2: Temporal analysis of STI prevalence in WSMW, WSWO and WSMO attending the Melbourne Sexual Health Centre from 2011-2019***

| **STI** | **Population** | **2011**  **n/N (%)** | **2012**  **n/N (%)** | **2013**  **n/N (%)** | **2014**  **n/N (%)** | **2015**  **n/N (%)** | **2016**  **n/N (%)** | **2017**  **n/N (%)** | **2018**  **n/N (%)** | **2019**  **n/N (%)** | **P_trend_ for 2011-2019** | **P_trend_ for 2011-2014** | **P_trend_ for 2015-2019** |
| --- | --- | --- | --- | --- | --- | --- | --- | --- | --- | --- | --- | --- | --- |
| **Bacterial Vaginosis** | *WSMW* | 19/128 (14.8) | 16/191 (8.4) | 22/200 (11.0) | 30/239 (12.6) | 30/261 (11.5) | 33/344 (9.6) | 46/362 (12.7) | 53/369 (14.4) | 60/524 (11.5) | 0.532 |  |  |
|  | *WSWO* | 9/55 (16.4) | 6/49 (12.2) | 12/63 (19.0) | 9/64 (14.1) | 9/52 (17.3) | 6/50 (12.0) | 5/50 (10.0) | 13/74 (17.6) | 10/77 (13.0) | 0.640 |  |  |
|  | *WSMO* | 170/2416 (7.0) | 170/2892 (5.9) | 228/3398 (6.7) | 303/3501 (8.7) | 278/3649 (7.6) | 308/3949 (7.8) | 325/3769 (8.6) | 365/4443 (8.2) | 393/4978 (7.9) | <0.001 (↑)^[[1]](#footnote-1)^ |  |  |
| **Candidiasis** | *WSMW* | 10/128 (7.8) | 13/191 (6.8) | 15/200 (7.5) | 24/239 (10.0) | 19/261 (7.3) | 22/344 (6.4) | 30/362 (8.3) | 30/369 (8.1) | 47/524 (9.0) | 0.507 |  |  |
|  | *WSWO* | 2/55 (3.6) | 4/49 (8.2) | 5/63 (7.9) | 4/64 (6.3) | 8/52 (15.4) | 4/50 (8.0) | 3/50 (6.0) | 7/74 (9.5) | 4/77 (5.2) | 0.828 |  |  |
|  | *WSMO* | 216/2416 (8.9) | 228/2892 (7.9) | 283/3398 (8.3) | 314/3501 (9.0) | 307/3649 (8.4) | 328/3949 (8.3) | 355/3769 (9.4) | 370/4443 (8.3) | 485/4978 (9.7) | 0.053 |  |  |
| **HSV** | *WSMW* | 8/128 (6.3) | 5/191 (2.6) | 9/200 (4.5) | 3/239 (1.3) | 9/261 (3.4) | 11/344 (3.2) | 15/362 (4.1) | 14/369 (3.8) | 10/524 (1.9) | 0.228 |  |  |
|  | *WSWO* | 3/55 (5.5) | 3/49 (6.1) | 2/63 (3.2) | 1/64 (1.6) | 2/52 (3.8) | 1/50 (2.0) | 0/50 (0.0) | 3/74 (4.1) | 4/77 (5.2) | 0.700 |  |  |
|  | *WSMO* | 96/2416 (4.0) | 105/2892 (3.6) | 117/3398 (3.4) | 119/3501 (3.4) | 109/3649 (3.0) | 127/3949 (3.2) | 103/3769 (2.7) | 152/4443 (3.4) | 143/4978 (2.9) | 0.009 (↓)^[[2]](#footnote-2)^ |  |  |
| **PID** | *WSMW* | 5/128 (3.9) | 5/191 (2.6) | 4/200 (2.0) | 4/239 (1.7) | 5/261 (1.9) | 9/344 (2.6) | 10/362 (2.8) | 7/369 (1.9) | 16/524 (3.1) | 0.848 |  |  |
|  | *WSWO* | 0/55 (0.0) | 0/49 (0.0) | 0/63 (0.0) | 0/64 (0.0) | 0/52 (0.0) | 0/50 (0.0) | 1/50 (2.0) | 0/74 (0.0) | 1/77 (1.3) | 0.139 |  |  |
|  | *WSMO* | 60/2416 (2.5) | 78/2892 (2.7) | 71/3398 (2.1) | 98/3501 (2.8) | 94/3649 (2.6) | 93/3949 (2.4) | 126/3769 (3.3) | 117/4443 (2.6) | 95/4978 (1.9) | 0.545 |  |  |
| **Chlamydia** | *WSMW* | 12/125 (9.6) | 9/176 (5.1) | 19/192 (9.9) | 14/235 (6.0) | 12/250 (4.8) | 19/333 (5.7) | 25/354 (7.1) | 22/360 (6.1) | 35/509 (6.9) | 0.570 |  |  |
|  | *WSWO* | 0/47 (0.0) | 0/42 (0.0) | 0/55 (0.0) | 0/56 (0.0) | 0/47 (0.0) | 1/49 (2.0) | 0/46 (0.0) | 3/72 (4.2) | 2/74 (2.7) | 0.014 (↑) |  |  |
|  | *WSMO* | 195/2181 (8.9) | 216/2597 (8.3) | 288/3169 (9.1) | 317/3266 (9.7) | 293/3382 (8.7) | 343/3726 (9.2) | 358/3582 (10.0) | 399/4222 (9.5) | 442/4686 (9.4) | 0.109 |  |  |
| Gonorrhoea | **WSMW** | 0/69 (0.0) | 1/102 (1.0) | 0/103 (0.0) | 3/126 (2.4) | 2/120 (1.7) | 1/201 (0.5) | 5/287 (1.7) | 4/361 (1.1) | 4/509 (0.8) | 0.905 |  |  |
|  | **WSWO** | 0/24 (0.0) | 0/22 (0.0) | 0/29 (0.0) | 0/28 (0.0) | 0/26 (0.0) | 0/24 (0.0) | 1/34 (2.9) | 0/72 (0.0) | 0/74 (0.0) | 0.725 |  |  |
|  | **WSMO** | 8/1170 (0.7) | 9/1230 (0.7) | 8/1455 (0.5) | 16/1524 (1.0) | 27/1463 (1.8) | 29/1848 (1.6) | 44/2594 (1.7) | 54/4211 (1.3) | 43/4692 (0.9) | 0.137 |  |  |
| *Culture* | ***WSMW*** | *0/69 (0.0)* | *1/102 (1.0)* | *0/103 (0.0)* | *3/126 (2.4)* | *-* | *-* | *-* | *-* | *-* | *NA* | *0.147* | *NA* |
|  | ***WSWO*** | *0/24 (0.0)* | *0/22 (0.0)* | *0/29 (0.0)* | *0/28 (0.0)* | *-* | *-* | *-* | *-* | *-* | *NA* | *NA* | *NA* |
|  | ***WSMO*** | *8/1170 (0.7)* | *9/1230 (0.7)* | *8/1455 (0.5)* | *16/1524 (1.0)* | *-* | *-* | *-* | *-* | *-* | *NA* | *0.353* | *NA* |
| *NAAT +/- culture* | ***WSMW*** | *-* | *-* | *-* | *-* | *2/120 (1.7)* | *1/201 (0.5)* | *5/287 (1.7)* | *4/361 (1.1)* | *4/509 (0.8)* | *NA* | *NA* | *0.541* |
|  | ***WSWO*** | *-* | *-* | *-* | *-* | *0/26 (0.0)* | *0/24 (0.0)* | *1/34 (2.9)* | *0/72 (0.0)* | *0/74 (0.0)* | *NA* | *NA* | *0.637* |
|  | ***WSMO*** | *-* | *-* | *-* | *-* | *27/1463 (1.8)* | *29/1848 (1.6)* | *44/2594 (1.7)* | *54/4211 (1.3)* | *43/4692 (0.9)* | *NA* | *NA* | *0.001 (↓)* |
| Trichomonas | **WSMW** | 0/70 (0.0) | 1/95 (1.1) | 1/72 (1.4) | 1/70 (1.4) | 1/71 (1.4) | 0/94 (0.0) | 1/75 (1.3) | 0/80 (0.0) | 2/103 (1.9) | 0.679 |  |  |
|  | **WSWO** | 0/24 (0.0) | 0/21 (0.0) | 2/24 (8.3) | 0/17 (0.0) | 0/16 (0.0) | 0/9 (0.0) | 0/11 (0.0) | 0/14 (0.0) | 0/14 (0.0) | 0.459 |  |  |
|  | **WSMO** | 6/1171 (0.5) | 2/1158 (0.2) | 3/1063 (0.3) | 3/989 (0.3) | 4/876 (0.5) | 8/891 (0.9) | 5/804 (0.6) | 18/959 (1.9) | 22/1048 (2.1) | <0.001 (↑) |  |  |
| HIV | **WSMW** | 0/79 (0.0) | 0/121 (0.0) | 0/143 (0.0) | 0/167 (0.0) | 0/178 (0.0) | 0/202 (0.0) | 0/236 (0.0) | 0/283 (0.0) | 1/400 (0.3) | 0.240 |  |  |
|  | **WSWO** | 0/22 (0.0) | 0/17 (0.0) | 0/32 (0.0) | 0/22 (0.0) | 0/29 (0.0) | 0/24 (0.0) | 0/27 (0.0) | 0/40 (0.0) | 0/51 (0.0) | NA |  |  |
|  | **WSMO** | 5/1209 (0.4) | 8/1434 (0.6) | 6/1769 (0.3) | 5/1835 (0.3) | 10/1937 (0.5) | 8/2006 (0.4) | 7/2136 (0.3) | 12/3034 (0.4) | 6/3344 (0.2) | 0.150 |  |  |
| Syphilis | **WSMW** | 0/64 (0.0) | 0/119 (0.0) | 0/143 (0.0) | 0/168 (0.0) | 0/182 (0.0) | 0/199 (0.0) | 1/247 (0.4) | 2/286 (0.7) | 3/403 (0.7) | 0.028 (↑) |  |  |
|  | **WSWO** | 0/18 (0.0) | 0/17 (0.0) | 0/31 (0.0) | 0/23 (0.0) | 0/30 (0.0) | 0/25 (0.0) | 0/28 (0.0) | 0/42 (0.0) | 0/54 (0.0) | NA |  |  |
|  | **WSMO** | 12/995 (1.2) | 22/1448 (1.5) | 14/1797 (0.8) | 16/1861 (0.9) | 13/1966 (0.7) | 16/2044 (0.8) | 35/2191 (1.6) | 29/3078 (0.9) | 38/3435 (1.1) | 0.884 |  |  |

1. Upward arrows indicate an increase in the P_trend_ over time. [↑](#footnote-ref-1)
2. Downward arrows indicate a decrease in the P_trend_ over time. [↑](#footnote-ref-2)
